# Metformin Promotes Axon Regeneration after Spinal Cord Injury through Inhibiting Oxidative Stress and Stabilizing Microtubule

**DOI:** 10.1155/2020/9741369

**Published:** 2020-01-06

**Authors:** Haoli Wang, Zhilong Zheng, Wen Han, Yuan Yuan, Yao Li, Kailiang Zhou, Qingqing Wang, Ling Xie, Ke Xu, Hongyu Zhang, Huazi Xu, Yanqing Wu, Jian Xiao

**Affiliations:** ^1^Department of Orthopaedics, The Second Affiliated Hospital and Yuying Children's Hospital of Wenzhou Medical University, Wenzhou, Zhejiang, China; ^2^Molecular Pharmacology Research Center, School of Pharmaceutical Science, Wenzhou Medical University, Wenzhou, Zhejiang, China; ^3^The Institute of Life Sciences, Engineering Laboratory of Zhejiang Province for Pharmaceutical Development of Growth Factors, Biomedical Collaborative Innovation Center of Wenzhou, Wenzhou University, Wenzhou, Zhejiang, China 325035

## Abstract

Spinal cord injury (SCI) is a devastating disease that may lead to lifelong disability. Thus, seeking for valid drugs that are beneficial to promoting axonal regrowth and elongation after SCI has gained wide attention. Metformin, a glucose-lowering agent, has been demonstrated to play roles in various central nervous system (CNS) disorders. However, the potential protective effect of metformin on nerve regeneration after SCI is still unclear. In this study, we found that the administration of metformin improved functional recovery after SCI through reducing neuronal cell apoptosis and repairing neurites by stabilizing microtubules via PI3K/Akt signaling pathway. Inhibiting the PI3K/Akt pathway with LY294002 partly reversed the therapeutic effects of metformin on SCI in vitro and vivo. Furthermore, metformin treatment weakened the excessive activation of oxidative stress and improved the mitochondrial function by activating the nuclear factor erythroid-related factor 2 (Nrf2) transcription and binding to the antioxidant response element (ARE). Moreover, treatment with Nrf2 inhibitor ML385 partially abolished its antioxidant effect. We also found that the Nrf2 transcription was partially reduced by LY294002 in vitro. Taken together, these results revealed that the role of metformin in nerve regeneration after SCI was probably related to stabilization of microtubules and inhibition of the excessive activation of Akt-mediated Nrf2/ARE pathway-regulated oxidative stress and mitochondrial dysfunction. Overall, our present study suggests that metformin administration may provide a potential therapy for SCI.

## 1. Introduction

Traumatic spinal cord injury (SCI) is one of the major cause of public health problems in the world. Millions of people suffer from neurological complications related to SCI, including quadriplegia or paraplegia [1, 2]. SCI results in neurological deficits after primary and secondary injury. The primary injury causes a structural disturbance at the time of injury [3]. Then, a long-term secondary injury is considered a complicated and multifactorial stage that can cause a series of detrimental effects, including oxidative stress, inflammation, and mitochondrial dysfunction, which eventually contributes to neuronal apoptosis and inhibits axon regeneration and nerve recovery [4–6]. Therefore, effective prevention of detrimental secondary events by reduction of neuronal cell death and promotion of axon regeneration is a potential approach for improving functional recovery after SCI.

It is well known that the secondary injury caused by SCI induces neuronal cell death [7]. Furthermore, this neuronal cell death largely results in injured axons, which is difficult for them to regenerate and reestablish connections with the other neurons during injury [8]. During axon formation, microtubule assembly is crucial for neuronal polarization and axonal growth [9, 10]. Increasing microtubule stabilization prevents swelling of the axon tip and axonal retraction after CNS injury, thus promoting the axonal growth of cultured neurons [11]. Recently, some studies have demonstrated that pharmacological treatment can boost axon growth and enhance axon regeneration by increasing microtubule stabilization [12]. Moreover, it was reported that FGF13 stabilizes microtubules and improves mitochondrial function in order to enhance axon regeneration after SCI [13]. Therefore, regulating microtubule stabilization to regenerate axons is considered as a therapeutic approach for SCI.

Oxidative stress, a highly disordered metabolic process, is the result of an imbalance between antioxidant and prooxidant [14]. Recent studies have demonstrated that oxidative stress is involved in a range of neurological diseases, including neurodegeneration disorders, cerebral ischemia, and SCI [15–17]. Previous studies have revealed that the reactive oxygen species (ROS) production, which is one of the major detrimental effects during secondary injury, showed a significant increase after SCI [18]. Once the spinal cord suffered from damage, the lesion site is accompanied with hypoxia-ischemia and inflammation and results in a redundant production of ROS. Therefore, the prevention of oxidative stress development and accumulation of ROS using antioxidants could be a helpful for SCI recovery. A previous study has suggested that antioxidant treatments can trigger the increase of stable microtubules and promote axonal regrowth [19], but the role of oxidative stress in microtubule stabilization after SCI remains unclear. In the antioxidant defensive system, the nuclear factor erythroid 2-related factor 2 (Nrf2) binds to the antioxidant response element (ARE), a cis-acting regulatory element of genes encoding antioxidant proteins and phase II detoxification enzymes, thereby regulating the expression of a large group of cytoprotective genes such as heme oxygenase-1 (HO-1) and NADH dehydrogenase quinone 1 (NQO1) that is involved in the cellular antioxidant responses [20, 21]. As one of upstream signal molecule for regulating Nrf2, the PI3K/Akt pathway is critical for the survival and growth, which participates in many biological processes such as cytoskeletal dynamics and antiapoptosis [22]. In addition, the PI3K/Akt pathway regulates the Nrf2/ARE pathway and then inhibits oxidative stress and inflammation after SCI [23]. Thus, activating the Nrf2/ARE signaling pathway is implicated as a potential therapy for the reduction of oxidative stress and promotion of functional recovery after SCI.

Metformin, as a traditional hypoglycemic agent, is widely prescribed for the treatment of type 2 diabetes and other metabolic syndromes since 1960s [24]. Metformin ameliorates hyperglycemia via inhibiting hepatic glucose production and increasing peripheral glucose utilization [25]. However, its ability is not limited to lowering glucose. Accumulating evidence suggests that metformin has benefits in various central nervous system (CNS) disorders, including ischemic brain injury, Parkinson's disease, and Huntington's disease [26–28]. In rat brain microvascular endothelial cells (RBEC), metformin increased transendothelial electrical resistance of RBEC monolayers and decreased sodium fluorescein and Evans Blue permeability via upregulating tight junction (TJ) proteins [29]. In addition, metformin has been shown to have a protective effect in improving mitochondrial homeostasis following oxidative stress-induced apoptosis in endothelial cells [30]. Recently, studies have shown that metformin treatment has improved locomotor recovery after SCI [31, 32], but the underlying mechanism of its action is still unclear, especially its role in reducing oxidative stress or promoting axonal regeneration after SCI. Moreover, the other studies have indicated that metformin can protect against hypoxic-ischemic brain injury-induced neuronal cell apoptosis [33]. However, it is unknown whether metformin protects against neuronal damage after SCI by promoting axonal regeneration. Many studies have reported that metformin is associated with the PI3K/Akt signaling pathway. Metformin was found to suppress the PI3K/Akt pathway in the treatment of esophageal cancer cell [34]. Another study have reported that the effect of metformin on ischemic heart was mediated through the PI3K/Akt pathway [35]. However, it is unclear whether the PI3K/Akt signaling pathway is involved in the role of metformin in SCI.

In the present study, we systematically investigated the role of metformin in antioxidant and neuronal regeneration after SCI. We have found some of the possible molecular mechanisms underlying SCI via the promotion of microtubule stabilization and reduction of apoptosis by activating the PI3K/Akt pathway. Moreover, this neuroprotective effect of metformin was also related to the inhibition of excessive oxidative stress and improvement of mitochondrial function by activating the Nrf2/ARE pathway. These findings have revealed that metformin administration promotes the recovery of SCI and suggested that metformin has the potential to be used in the clinical treatment of SCI.

## 2. Material and Methods

### 2.1. Spinal Cord Injury and Drug Treatment

Sprague-Dawley rats (female; 220-250 g, *N* = 80) were purchased from Animal Center of Chinese Academy of Science. The animals were housed in standard temperature conditions with a 12 h light/dark cycle and regularly fed with food and water. After anesthetizing with 2% pentobarbital sodium (40 mg/kg, i.p.), rats were performed with a laminectomy at the T9 level exposing the cord beneath without disrupting the dura. Then, rats suffered with a compression of a vascular clip (15 g forces, Oscar, China) for 1 minute. For the sham control group, the rats received the same surgical procedure but no impact injury and received no pharmacological treatment. Metformin was diluted with normal saline, to achieve a final metformin concentration of 20 mg/mL. After surgery, rats were given metformin solution (50 mg/kg) with/without LY294002 (a specific PI3K inhibitor, 1.2 mg/kg) immediately via i.p. injection and then were injected with the same dose of metformin solution with/without LY294002 per day until the rats were sacrificed. Rats in SCI groups received equivolumetric injection of normal saline at the corresponding time points after injury. Postoperative care included manual urinary bladder emptying twice daily until the return of bladder function and the administration of cefazolin sodium (50 mg/kg, i.p.). Following completion of the trial, rats were euthanized using an overdose of pentobarbital sodium on 7 d and 14 d, with the exception of 24 rats for locomotion recovery assessment. All surgical interventions and postoperative animal care were approved by the ethics committee of Wenzhou Medical University and performed in accordance with the *Guide for the Care and Use of Laboratory Animals*.

### 2.2. Locomotion Recovery Assessment

The Basso, Beattie, and Bresnahan (BBB) scores were assessed by three trained investigators who were blinded to experiment in an open-field scale at 1, 3, 5, 7, and 14 d postoperation. Briefly, the BBB scores range from 0 points (complete paralysis) to 21 points (normal locomotion). The scale was developed using the natural progression of locomotion recovery in rats with thoracic SCI [36]. Moreover, the footprint analysis was performed by dipping the rat's fore limb with blue dye and the posterior limb with red dye at 14 d after SCI. Ten rats for each group were used to assess the motor function.

### 2.3. Hematoxylin and Eosin Staining and Nissl Staining

The rats of each group (*n* = 5) were euthanized with an overdose of sodium pentobarbital, followed by 4% paraformaldehyde in 0.01 M phosphate-buffered saline (PBS, pH = 7.4) at 7 d after SCI. Tissue segments containing the lesion (1 cm on each side of the lesion) were paraffin embedded. Transverse paraffin sections (5 *μ*m thick) were mounted on poly-L-lysine-coated slides for hematoxylin and eosin (H&E) staining and Nissl staining and examined under a light microscope. The cellular stain HE was used to observe the cavity, at 5 mm from the lesion site. The measurements were reported as the percentage of the preserved area in relation to the total area of each section analyzed [37]. For Nissl staining, the number of ventral motor neuron (VMN) in sections was assessed as in the previous report [38]. Transverse sections were collected at rostral, caudal 5 mm, and the lesion site and stained with cresyl violet acetate. After determination of the cells located in the lower ventral horn, cells larger than half of the sampling square (20 × 20 *μ*m) were counted as a VMN. The cells above the line at 150 *μ*m ventral from the central canal were excluded. The cells were manually counted from each field using MetaMorph software.

### 2.4. Cell Culture Treatment Protocols

The PC12 cells were purchased from Cell Bank of Type Culture Collection of Chinese Academy of Sciences, Shanghai Institute of Cell Biology, Chinese Academy of Sciences. PC12 cells were cultured in RPMI 1640 medium supplemented with 10% fetal bovine serum (FBS), 100 U/mL penicillin, and 100 U/mL streptomycin. Cells were incubated in a humidified atmosphere containing 5% CO_2_ and 95% air at 37°C. PC12 cells were treated with metformin (1 mM), LY294002 (10 *μ*M), ML385 (a novel and specific Nrf2 inhibitor, 2 *μ*M), and H_2_O_2_ (100 *μ*M) for 8 h. All experiments were performed at least three times.

### 2.5. Primary Cortical Neuron Culture

The primary cortical neurons were obtained from pregnant Sprague-Dawley rats with embryonic (E18) fetuses. Briefly, fetal rats were sacrificed by decapitation. The cerebral cortex was separated and rinsed in ice-cold Hank's buffer. After clearing up the blood vessels, the cortical tissues were cut into approximately 1 mm pieces and were then treated with 0.125% trypsin-EDTA for 20 min at 37°C. After incubation, the solution was filtered by a 100 *μ*m cell strainer (WHB). The cell suspension was centrifuged at 1000 rpm for 5 min, and the cell pellet was resuspended in complete DMEM/F-12. Cells were incubated for 4 h in 5% CO_2_ at 37°C. Then, the cells were refreshed with neuronal basal medium (Gibco, Invitrogen) containing 2% B27 and 0.5 mM L-glutamine (GlutaMAX™ Supplement, Gibco) and cultured in a humidified atmosphere of 5% CO_2_ and 95% air at 37°C. The medium was replaced every 3 d.

### 2.6. Western Blotting Analysis

Spinal cord tissue samples were extracted 7 d and 14 d after surgery and immediately snap-frozen at -80°C for western blotting. Briefly, the tissues were lysed using RIPA buffer (1% Triton X-100, 0.5% sodium deoxycholate, 1 mM PMSF, 1 mM EDTA, 10 *μ*g/mL leupeptin, 20 mM Tris-HCl, 150 mM NaCl, and pH 7.5). In vitro, the cells were rinsed twice with PBS and lysed in lysis buffer (1% Nonidet P-40, 0.1% SDS, 1% sodium deoxycholate, 25 mM Tris-HCl, 150 mM NaCl, and pH 7.6). Protein extraction of both the cytosolic and nuclear was performed by using the Nuclear and Cytoplasmic Protein Extraction Kit (P0027, Beyotime, China) according to the manufacturer's protocol. Tissue and cell lysates were centrifuged at 12,000 rpm for 10 min at 4°C, and the supernatant was obtained for a protein assay. Protein concentrations were quantified with the Enhanced BCA Protein Assay Kit (Beyotime, Shanghai, China). 40 *μ*g of tissue protein was separated by SDS-PAGE and transferred to a PDGF membrane (Bio-Rad, California, USA). After blocking with 5% nonfat milk (Bio-Rad) for 2 h, the membranes were then incubated with the primary antibody against GAPDH (1 : 10000, Bioworld), phosphor-Akt (1 : 1000, Abcam), total-Akt (1 : 1000, Abcam), cleaved caspase3 (1 : 500, Abcam), Bax (1 : 1000, Cell Signaling Technology), Bcl-2 (1 : 1000, Abcam), Ace-tubulin (1 : 1000, Cell Signaling Technology), Tyr-tubulin (1 : 1000, Sigma), MAP2 (1 : 1000, Cell Signaling Technology), GAP43 (1 : 1000, Abcam), Nrf2 (1 : 1000, Abcam), NQO1 (1 : 1000, Abcam), HO-1 (1 : 10000, Abcam), and histone (1 : 1000, Abcam) at 4°C overnight. The membranes were washed with TBST (Tris-buffered saline with 0.1% Tween-20) three times and incubated with the secondary antibodies for 1 h at room temperature. Signals were visualized by a ChemiDoc XRS+ Imaging System (Bio-Rad). We analyzed the bands by using the Quantity One software.

### 2.7. Immunofluorescence Staining

Spinal cord tissue samples were obtained 7 d and 14 d after injury. All spinal cords were postfixed in 4% PFA, washed, and embedded in paraffin. Transverse sections of 5 *μ*m thickness were cut, deparaffinized in xylene, and rehydrated by ethanol washes. In addition, the sections were incubated with 10% normal goat serum for 1 h at room temperature in PBS containing 0.1% Triton X-100. They were then incubated with the appropriate primary antibodies overnight at 4°C in the same buffer. The following primary antibodies were used, based on differing targets: Ace-tubulin (1 : 500, Cell Signaling Technology), GFAP (1 : 500, Santa Cruz), NeuN (1 : 500, Abcam), GAP43 (1 : 500, Abcam), HO-1 (1 : 200, Abcam), NQO1 (1 : 1000, Abcam), and Tyr-tubulin (1 : 500, Sigma). After primary antibody incubation, sections were washed with PBST for 4 × 10 minutes and then incubated with Alexa Fluor 488/594 goat anti-rabbit/mouse secondary antibodies for 1 h at room temperature. Sections were rinsed three times with PBST and incubated with 4′,6-diamidino-2-phenylindole (DAPI) for 10 minutes and finally washed in PBST and sealed with a coverslip. For staining of F-actin, a rhodamine-coupled phalloidin was used (Yeasen, Shanghai). The images were captured with a confocal fluorescence microscope (Nikon, A1 PLUS, Tokyo, Japan); positive neurons in each section were counted by three observers who were blinded to the experimental groups. The rates of the corresponding protein-positive cells per section were calculated from values obtained by counting 30-40 random sections throughout the lesion site of each animal, with five animals examined per group.

### 2.8. TUNEL Assay

DNA fragmentation was detected using an *In Situ* Cell Death Detection Kit (Roche, South Francisco, CA, USA), and TUNEL staining was performed 7 d after SCI. The sections (5 *μ*m thick) were deparaffinized and rehydrated. Then, these sections were treated with a 20 *μ*g/mL proteinase K working solution for 20 min at 37°C. The sections were rinsed three times in PBS and incubated with TUNEL reaction mixture in a dark humidified box for 1 h at room temperature. Afterward, the sections were washed with PBS and treated with DAPI for 10 min at room temperature. For a positive control, spinal cord slices were treated with 10 U/mL DNase I buffer for 10 min at room temperature before incubation with the TUNEL reaction mixture. Negative control was obtained with the TUNEL reagent without the TdT enzyme. Positive cells were observed under a confocal fluorescence microscope (Nikon, A1 PLUS, Tokyo, Japan) and analyzed by using ImageJ software.

### 2.9. Measurement of Intracellular ROS Generation

An intracellular ROS level was detected by using the Reactive Oxygen Species Assay Kit (S0033, Beyotime, China). Briefly, according to the manufacturer's instructions, the PC12 cells were exposed to the H_2_O_2_ with or without metformin for 8 h and then stained with 10 mmol/L DCFH-DA for 30 min at 37°C. ROS levels were assessed through observation by a confocal fluorescence microscope in cells stained with DCFH-DA, and the intensity of fluorescence was measured and subjected to statistical analysis. For each sample, 20,000 cells were collected.

### 2.10. Measurement of ΔΨ*m* and ATP Levels

Mitochondrial membrane potential (ΔΨ*m*) was detected by using the JC-1 (C2006, Beyotime, China). PC12 cells were plated in confocal dished and incubated with culture medium containing JC-1 for 20 minutes at 37°C. Then, the cells were rinsed with ice-cold PBS twice, changed by fresh medium, and detected by using a confocal fluorescence microscope (Nikon, A1 PLUS, Tokyo, Japan).

ATP levels were detected using the ATP assay Kit (S0026, Beyotime, China) according to the manufacturer's protocol.

### 2.11. Statistical Analysis

The results were presented as mean ± standard error of the mean (SEM) from at least three independent experiments. Data were analyzed by one-way analysis of variance (ANOVA) followed by Dunnett's *post hoc* test for comparison between control and treatment groups. *P* < 0.05 was considered to indicate statistical significance.

## 3. Results

### 3.1. Metformin Decreases Spinal Cord Tissue Damage and Improves Locomotor Function in SCI Rats

**Figure 1:**
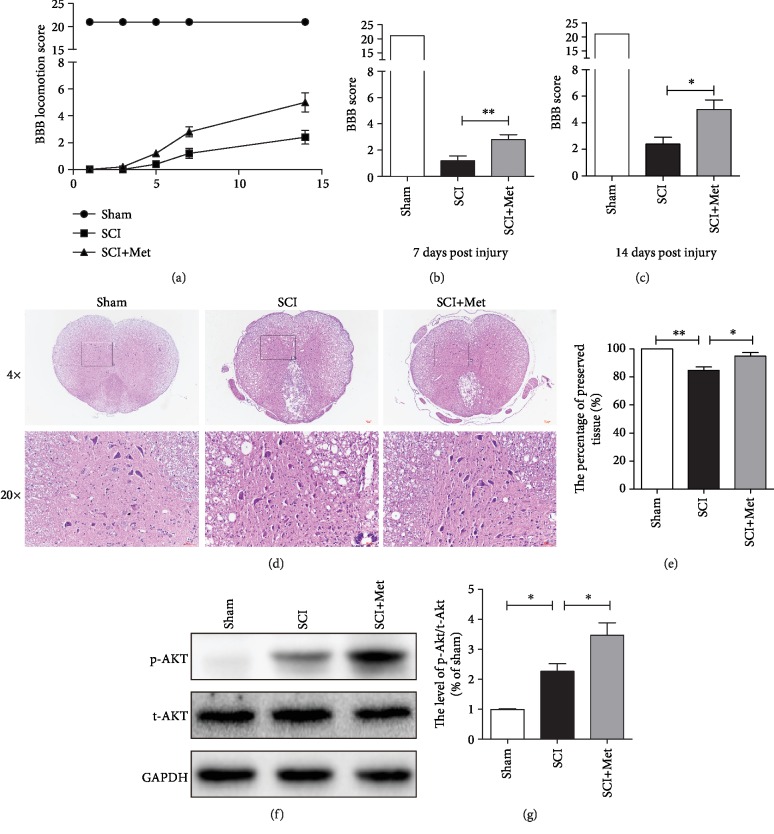
Metformin improved functional recovery after SCI. (a) The BBB locomotion scores of the different groups at 1, 3, 5, 7, and 14 days after SCI, *n* = 10 per group. (b, c) Quantification of the BBB locomotion scores at 7 and 14 d from (a). *n* = 10; ^∗^*P* < 0.05 and ^∗∗^*P* < 0.01*vs.* the indicated group. (d) Representative images from H&E at 7 dpi. Scale bar: 1000 *μ*m (4x). Scale bar: 200 *μ*m (20x). (e) Quantification data of the percentage of the preserved tissue area from (d). *n* = 5; ^∗^*P* < 0.05 and ^∗∗^*P* < 0.01*vs.* the indicated group. (f) Representative western blots of phosphor-Akt (p-Akt) and total-Akt (t-Akt) in each group. (g) Quantification of western blots data from (f). *n* = 5; ^∗^*P* < 0.05*vs.* the indicated group.

In this study, rats were given metformin solution (50 mg/kg, i.p.) immediately to determine whether the metformin has a potential to promote the recovery of SCI. A BBB rating scale were used to evaluate the therapeutic effect of metformin after SCI. As shown in Figure 1(a), the sham group performs slightly above of 20 units on the BBB locomotion score and untreated injured rats do at 1.5 (7 days) and 2 (14 days), the rescue on metformin-treated rats (score at around 3 and 4, respectively) is around 10% of the maximum achievable (Figures 1(a)–1(c)), indicating that locomotor function of the metformin group was remarkably improved when compared to the SCI group. Additionally, using H&E staining, we have further observed the damage of peripheral white matter and central gray matter after SCI. Consistent with the locomotion evaluation, the metformin-treated group showed less damage in the lesion area and preserved the motor neurons in the anterior horns (Figures 1(d) and 1(e)), indicating that metformin protected against severe damage during SCI. Meanwhile, some reports have confirmed that the PI3K/Akt signaling pathway is involved in the effect of metformin during ischemic heart [35]. Then, we have detected the phosphorylation status of Akt after SCI. The western blotting results have showed that the p-Akt was significantly upregulated after metformin treatment when compared with that in the SCI group (Figures 1(f) and 1(g)). These results suggested that the PI3K/Akt signaling pathway may be involved in the protective effect of metformin after SCI. The above findings further indicate that metformin has a neuroprotective efficacy on motor neurons during SCI.

### 3.2. Activation of the PI3K/Akt Signaling Pathway Is Involved in the Effect of Metformin after SCI

**Figure 2:**
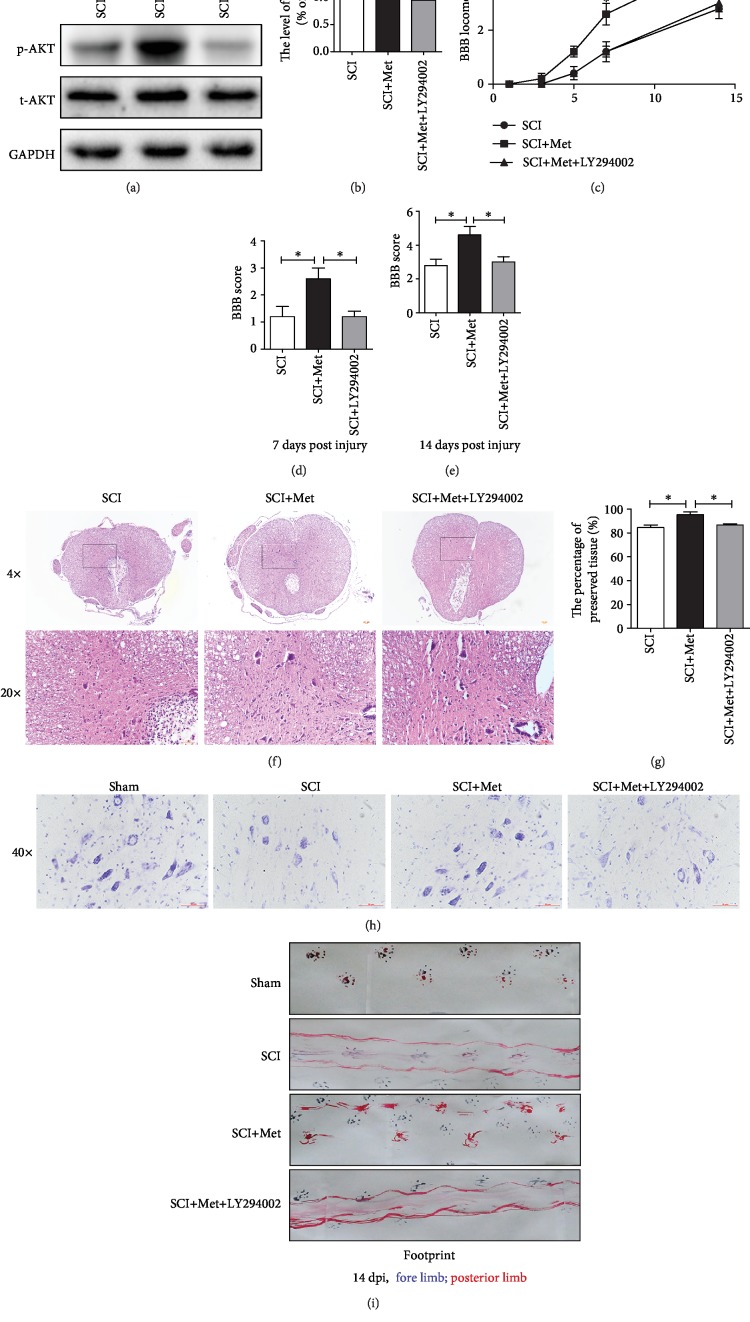
Metformin attenuated spinal cord tissue damage and motor neuron death and promoted functional recovery by activating the PI3K/Akt signaling pathway. (a) Representative western blots of phosphor-Akt (p-Akt) and total-Akt (t-Akt) in each group. (b) Quantification of western blots data from (a). *n* = 5; ^∗^*P* < 0.05 and ^∗∗^*P* < 0.01*vs.* the indicated group. (c) The BBB locomotion scores of the different groups at 1, 3, 5, 7, and 14 d after SCI, *n* = 10 per group. (d, e) Quantification of the BBB locomotion scores at 7 and 14 d from (c). *n* = 10; ^∗^*P* < 0.05*vs.* the indicated group. (f) Representative images from H&E at 7 dpi. Scale bar: 1000 *μ*m (4x). Scale bar: 200 *μ*m (20x). (g) Quantification data of the percentage of the preserved tissue area from (f). *n* = 5; ^∗^*P* < 0.05*vs.* the indicated group. (h) Nissl staining of each group to test the surviving neurons at 14 d after SCI. (i) Footprint analysis results from the different groups.

To further evaluate whether activating the PI3K/Akt signaling pathway is essential for metformin promoting the recovery of SCI, LY294002 (a specific PI3K inhibitor) was used to inhibit the PI3K/Akt signaling pathway. It was observed that p-Akt was significantly increased after metformin treatment when compared with that in the SCI group, and these increase was markedly inhibited by LY294002 treatment (Figures 2(a) and 2(b)). As shown in Figures 2(c)–2(e), BBB scores have also indicated that the protective effect of metformin on functional recovery of SCI was markedly suppressed by LY294002 treatment. H&E staining has further revealed that LY294002 treatment significantly enlarged the lesion area when compared with that in metformin treatment alone group (Figures 2(f) and 2(g)). Moreover, LY294002 administration remarkably have reduced the motor neuron survival when compare to that in metformin treatment alone (Figure 2(h)). In footprint analysis, metformin-treated rats displayed coordinated crawling of posterior limb and very little toe dragging at 14 dpi. In contrast, the rats from SCI and LY294002 groups still showed uncoordinated crawling and extensive dragging (Figure 2(i)). The above results have demonstrated that metformin regulated the PI3K/Akt signaling pathway, increased neuronal survival, and finally promoted the functional recovery of SCI.

### 3.3. Metformin Reduces the Apoptosis through Activation of the PI3K/Akt Signaling Pathway

**Figure 3:**
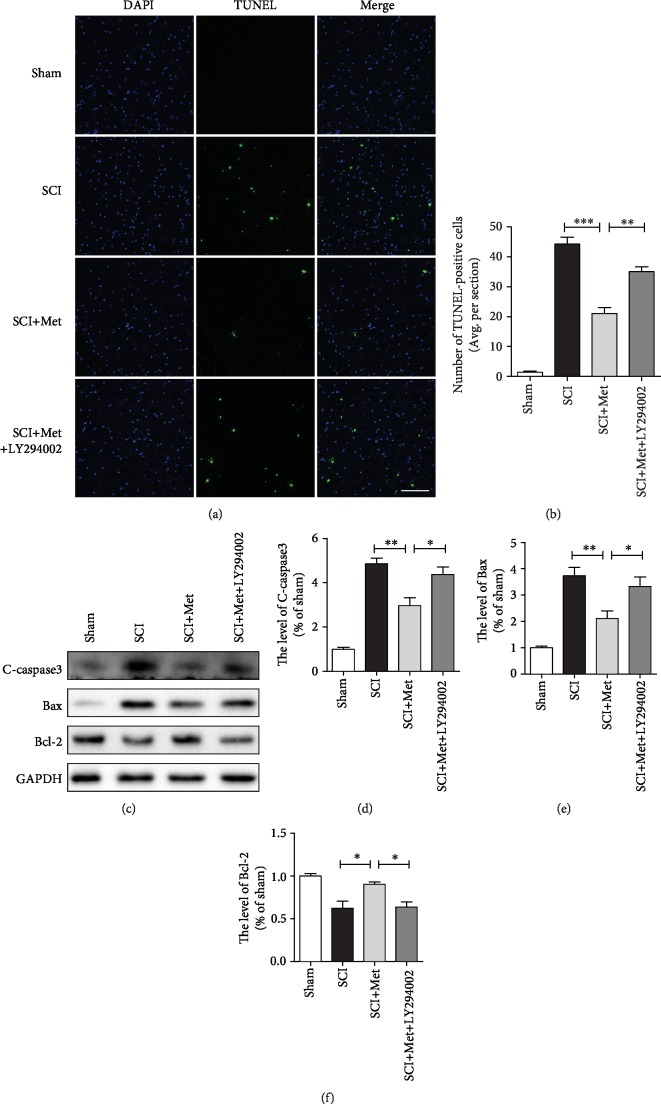
Metformin treatment blocked SCI-triggered apoptosis via activating the PI3K/Akt pathway after SCI. (a, b) TUNEL staining in the sham, SCI, SCI+metformin, and SCI+metformin+LY294002 group. Scale bar = 100 *μ*m. (c) Representative western blots of cleaved caspase3 (C-caspase3), Bax, and Bcl-2 in each group. (d–f) Quantification of western blots data from (c). *n* = 5; ^∗^*P* < 0.05 and ^∗∗^*P* < 0.01*vs.* the indicated group.

TUNEL staining was performed to evaluate whether metformin treatment reduces the apoptosis level after SCI. It was found that SCI dramatically increased the number of apoptotic cells, and metformin treatment ameliorated it. However, this protective effect of metformin was partly weakened by LY294002 treatment (Figures 3(a) and 3(b)). Moreover, consistent with TUNEL, western blot analysis has also showed that metformin treatment markedly blocked SCI-induced increases of cleaved caspase3 and Bax expression. In contrast, metformin increased the level of Bcl-2 expression compared to that in the SCI group. However, this antiapoptotic effect of metformin was significantly reversed by LY294002 treatment (Figures 3(c)–3(f)). Taken together, the above results have further confirmed the antiapoptotic effect of metformin after SCI.

### 3.4. Metformin Promotes Neurite Reparation after SCI

**Figure 4:**
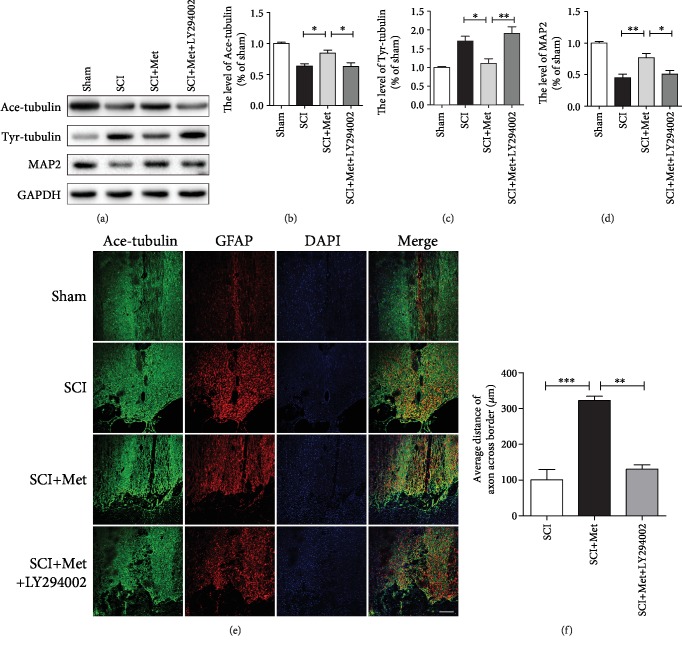
Metformin promoted neurite repair by activating the PI3K/Akt signaling pathway in acute SCI. (a) Representative western blots of Ace-tubulin, Tyr-tubulin, and MAP2 in each group. (b–d) Quantification of western blots data from (a). *n* = 5; ^∗^*P* < 0.05 and ^∗∗^*P* < 0.01*vs.* the indicated group. (e) Coimmunofluorescence images show Ace-tubulin (green) and GFAP (red) at 14 d after SCI in each group. Scale bar = 200 *μ*m. (f) Quantification of distance of axon across border. *n* = 5; ^∗∗^*P* < 0.01 and ^∗∗∗^*P* < 0.001*vs.* the indicated group.

**Figure 5:**
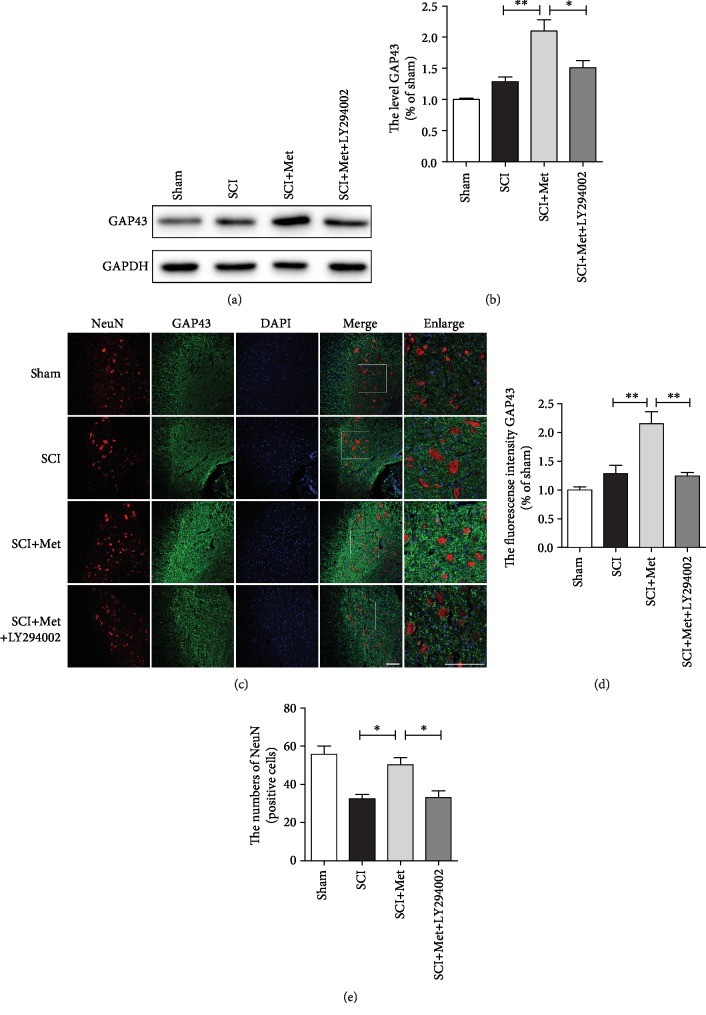
Metformin protects the neurons after SCI. (a) Representative western blots of GAP43 in each group. (b) Quantification of western blots data from (a). *n* = 5; ^∗^*P* < 0.05 and ^∗∗^*P* < 0.01*vs.* the indicated group. (c) Coimmunofluorescence images show GAP43 (green) and NeuN (red) after SCI in each group. Scale bar = 100 *μ*m. (d) Quantification of the fluorescence intensity (GAP43 level) from (c). ^∗∗^*P* < 0.01*vs.* indicated group. (e) Quantification of fluorescence (the number of NeuN) from (c). *n* = 5; ^∗^*P* < 0.05*vs.* the indicated group.

As shown above, metformin treatment exerted neuroprotective effect after SCI. Next, we have further explored whether metformin promotes axonal reparation. We examined the expression of acetylated-tubulin (Ace-tubulin; stable microtubules in axon), tyrosinated-tubulin (Tyr-tubulin; dynamic microtubules in axon), and microtubule-associated protein 2 (MAP2, a specific structural protein in neuronal dendrites) in each group at 14 dpi after SCI [39–41]. Western blotting results have revealed that Ace-tubulin and MAP2 expressions were significantly increased in the metformin group when compared to that in the SCI group. In contrast, metformin treatment dramatically attenuated the level of Tyr-tubulin when compared to that in the SCI group. Moreover, LY294002 treatment reversed the effect of metformin evidencing with decreases of Ace-tubulin and MAP2 and increases of Tyr-tubulin (Figures 4(a)–4(d)). In addition, coimmunofluorescence of GFAP-labeled astrocytes and Ace-tubulin-labeled axon was performed at 14 dpi. The results have showed that the GFAP-positive astrocytes were accumulated along the lesion border after SCI and metformin treatment promoted the axonal outgrowth to cross the lesion border and elongate farther into the distal area when compared with that in SCI and LY294002 treatment group (Figures 4(e) and 4(f)). To further confirm the neuroprotection effect of metformin, we have examined the expression of GAP43, which is neuronal protective protein [42]. The results indicated that the expression of GAP43 was at a very low level in the sham, SCI, and LY294002 treatment groups, which was significantly increased after metformin treatment (Figures 5(a) and 5(b)). Additionally, we have performed the coimmunofluorescence staining of GAP43 and NeuN, and found that metformin markedly increased the expression of GAP43 and reduced the loss of neurons (Figures 5(c)–5(e)). These data indicate that the metformin-activated PI3K/Akt signaling pathway contributes to axon regeneration after SCI.

### 3.5. Metformin Reduces Oxidative Stress via Activating the Nrf2/ARE Signaling Pathways after SCI

**Figure 6:**
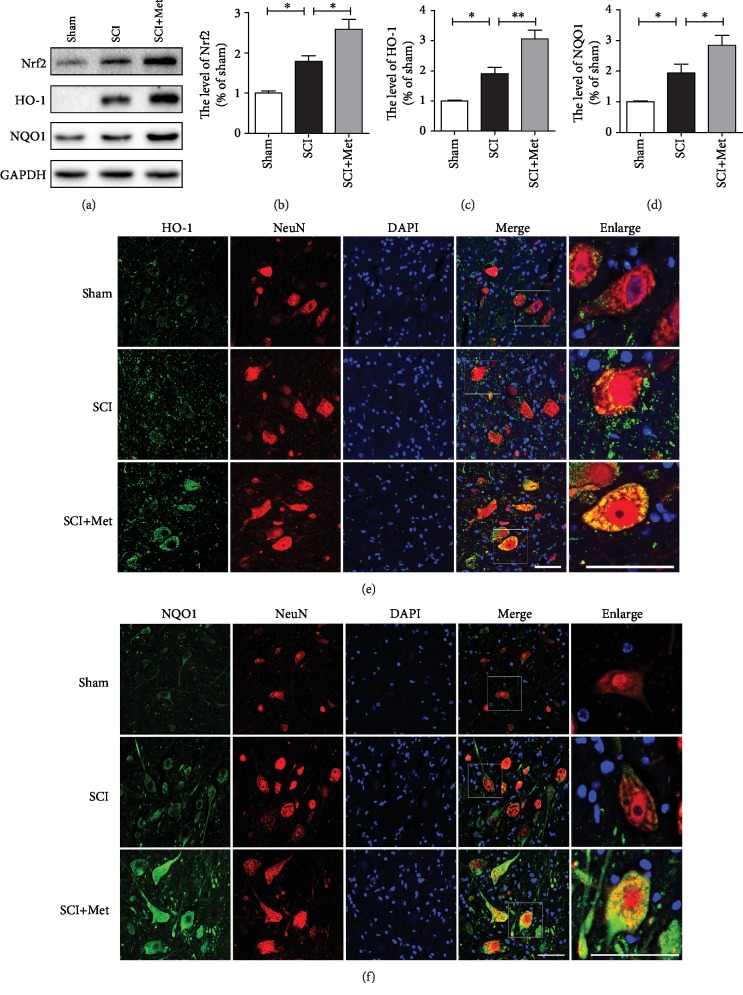
Metformin increased the expression of antioxidant proteins by activating the Nrf2/ARE signaling pathway after SCI. (a) Representative western blots of Nrf2, HO-1, and NQO1 in each group. (b–d) Quantification of western blots data from (a). *n* = 3; ^∗^*P* < 0.05 and ^∗∗^*P* < 0.01*vs.* the indicated group. (e) Coimmunofluorescence images show HO-1 (green) and NeuN (red) after SCI in each group. Scale bar = 50 *μ*m. (f) Coimmunofluorescence images show NQO1 (green) and NeuN (red) after SCI in each group. Scale bar = 50 *μ*m.

Lots of studies have demonstrated that the Nrf2/ARE signaling pathways are essential for the anti-inflammatory and antioxidant properties of metformin [43]. To further determine the mechanism underlying the therapeutic effect of metformin for SCI, we have evaluated whether the Nrf2/ARE signaling pathway is involved in the effect of metformin on SCI. We have detected the expression levels of Nrf2, HO-1, and NQO1. As shown in Figures 6(a)–6(d), the expressions of Nrf2, HO-1, and NQO1 levels were increased after SCI when compared with that in the sham group, suggesting that SCI activated the Nrf2/ARE signaling pathway. Compared with the SCI group, metformin treatment significantly induced the higher levels of Nrf2, HO-1, and NQO1. Additionally, immunofluorescence staining has also showed that the expressions of HO-1 and NQO1 in the metformin group was remarkably increased (Figures 6(e) and 6(f)). The above results have demonstrated that metformin treatment promotes the antioxidant level through activating the Nrf2/ARE signaling pathway after SCI.

### 3.6. Metformin Promotes Axon Regeneration and Migration in Neurons via Affecting Microtubule Stabilization

**Figure 7:**
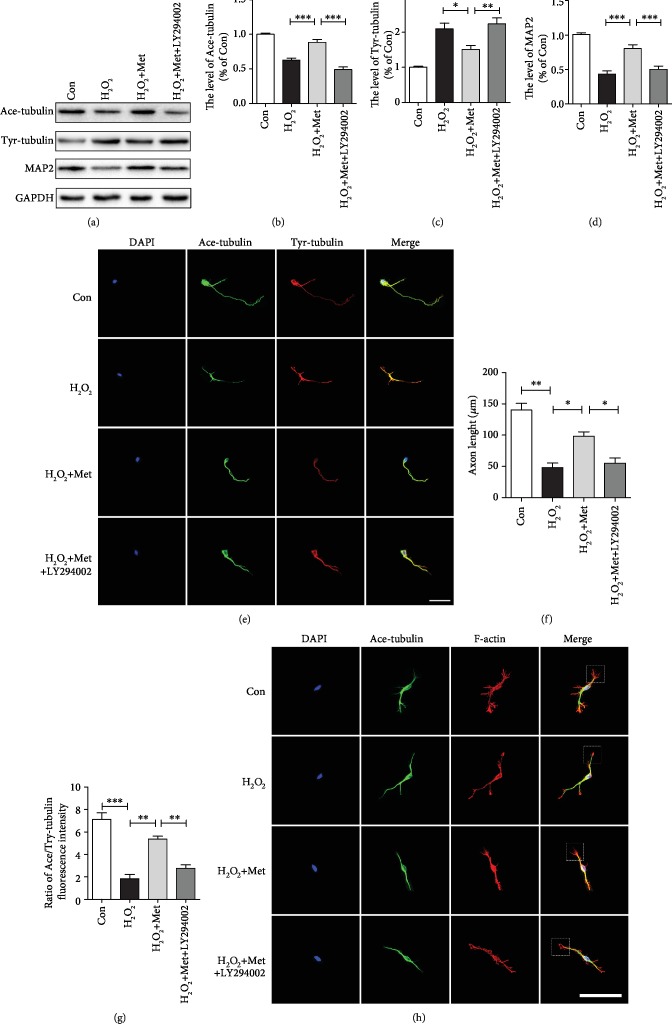
Metformin promote axonal regeneration by stabilizing microtubule in vitro. (a) Representative western blots of Ace-tubulin, Tyr-tubulin, and MAP2 in each group. (b–d) Quantification of western blots data from (a). ^∗^*P* < 0.05, ^∗∗^*P* < 0.01, and ^∗∗∗^*P* < 0.001*vs.* the indicated group. (e) Coimmunofluorescence images show Tyr-tubulin (green) and Ace-tubulin (red) in primary cortical neurons. Scale bar = 50 *μ*m. (f) Quantification of axonal length from (e). *n* = 4; ^∗^*P* < 0.05 and ^∗∗^*P* < 0.01*vs.* the indicated group. (g) Quantification of Ace-tubulin/Tyr-tubulin from (e). *n* = 4; ^∗∗^*P* < 0.01 and ^∗∗∗^*P* < 0.001*vs.* the indicated group. (h) Coimmunofluorescence images show growth cone (white frame) in primary cortical neurons. Scale bar = 100 *μ*m.

To further determine the effect of metformin, we have examined the expression levels of Ace-tubulin, Tyr-tubulin, and MAP2 in primary cortical neurons. In vitro, H_2_O_2_ treatment was used to stimulate the microcirculation of acute SCI. As shown in Figures 7(a)–7(d), western blotting results revealed that Ace-tubulin and MAP2 expressions were remarkably increased in neurons after metformin treatment when compared with those in the H_2_O_2_ group. In contrast, metformin significantly attenuated the level of Tyr-tubulin when compared to that in the H_2_O_2_ group. However, LY294002 treatment markedly blocked the effect of metformin on microtubule stabilization after SCI. Additionally, we have further evaluated the effect of metformin on microtubule stabilization in primary cortical neurons. The primary cortical neurons at DIV7 were provoked to H_2_O_2_ with and without metformin administration. Then, immunofluorescence staining was used to detect the expressions of Ace-tubulin and Tyr-tubulin. The results have indicated that the neurons without metformin treatment after H_2_O_2_ had a shorter axon than that of the control group. However, the axonal length in the metformin treatment group was remarkably longer than those in the H_2_O_2_ and LY294002 groups (Figures 7(e) and 7(f)). The ratio of Ace-tubulin to Tyr-tubulin was performed to evaluate the relative ratio of stable to dynamic microtubules [44]. We found that metformin administration caused a significant increase in the Ace-tubulin/Tyr-tubulin ratio compared to those in the H_2_O_2_ and LY294002 group (Figure 7(g)). We also used the primary cortical neurons at DIV3 to detect the shape of growth cone (white frame) by immunostaining. As shown in Figure 7(h), the mean size of growth cone was significantly increased in the metformin group when compared with those in the H_2_O_2_ and LY294002 groups. Therefore, above results have demonstrated that metformin can regulate microtubule stabilization and consequently increase the intrinsic growth ability of axon via activating the PI3K/Akt signaling pathway.

### 3.7. Metformin Alleviates Mitochondrial Dysfunction and Reduces ROS by Activating the Akt/Nrf2/ARE Signaling Pathway In Vitro

**Figure 8:**
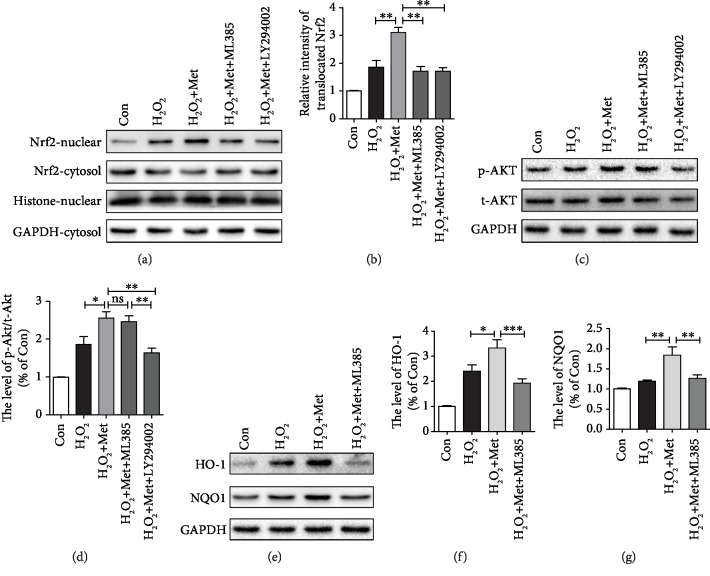
Metformin inhibited oxidative stress via activating the Akt/Nrf2/ARE signaling pathway. (a) Representative western blots of Nrf2-nuclear and Nrf2-cytosol in each group. (b) Quantification of western blots data from (a). ^∗∗^*P* < 0.01*vs.* the indicated group. (c) Representative western blots of p-Akt and t-Akt in each group. (d) Quantification of western blots data from (c). ^∗^*P* < 0.05 and ^∗∗∗^*P* < 0.001*vs.* the indicated group. (e) Representative western blots of HO-1 and NQO1 in each group. (f, g) Quantification of western blots data from (e). ^∗^*P* < 0.05, ^∗∗^*P* < 0.01, and ^∗∗∗^*P* < 0.001*vs.* the indicated group.

**Figure 9:**
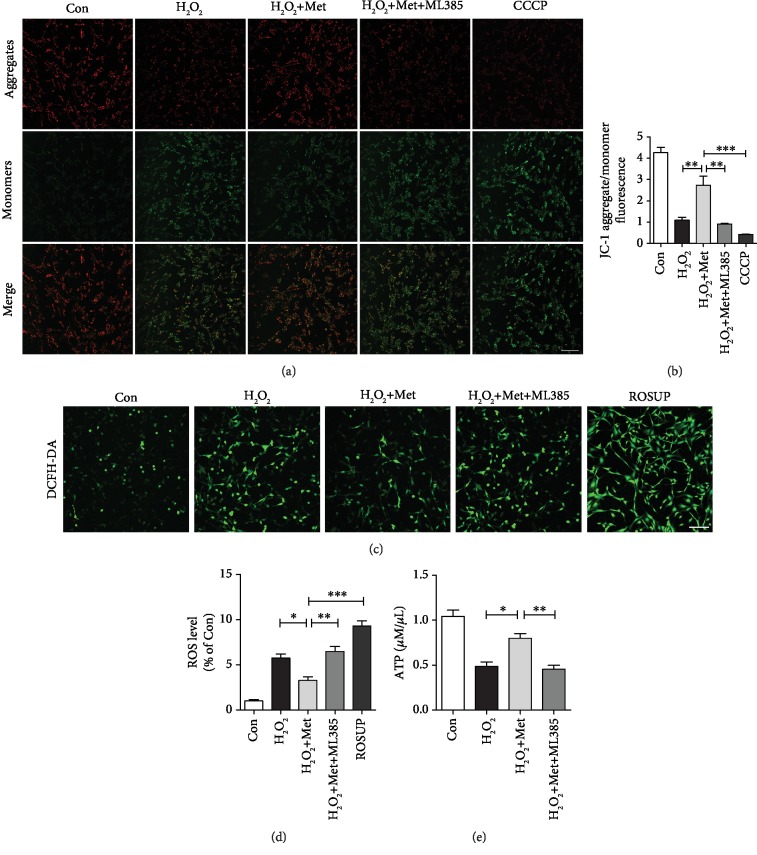
Metformin plays a protective role in mitochondrial function in vitro. (a) PC12 cells incubated with JC-1 represented the mitochondrial membrane potential in each group. Scale bar = 100 *μ*m. (b) Quantification of aggregate/monomer fluorescence ratio from (a). ^∗∗^*P* < 0.01 and ^∗∗∗^*P* < 0.001 vs. the indicated group. (c) The fluorescence images of the DCFH-DA probe for hydrogen peroxide of PC12 in each group. Scale bar = 100 *μ*m. (d) Quantification of fluorescence density from (c). ^∗^*P* < 0.05, ^∗∗^*P* < 0.01, and ^∗∗∗^*P* < 0.001 vs. the indicated group. (e) The levels of total ATP in each group. ^∗^*P* < 0.05 and ^∗∗^*P* < 0.01 vs. the indicated group.

Here, using H_2_O_2_-treated PC12 cells, we had further evaluated the effect of metformin treatment on antioxidant in vitro. We firstly detected the expression of Nrf2 protein. As shown in Figures 8(a) and 8(b), western blots of nuclear extracts with the anti-Nrf2 antibody showed that the level of translocated Nrf2 was increased after H_2_O_2_-treated and metformin significantly increased Nrf2 translocation. A previous study showed that Nrf2 is positively regulated by PI3K/Akt significantly and leads to the suppression of oxidative stress [23]. We used LY294002 and ML385 (a novel and specific Nrf2 inhibitor) [45, 46] to further confirm the role of Nrf2. The results showed that LY294002 treatment not only suppressed the p-Akt/t-Akt ratio, but also inhibited Nrf2 to translocate into the nucleus. However, ML385 only suppressed Nrf2 to the translocated nucleus with no obvious effect on the p-Akt expression (Figures 8(a)–8(d)). Similarly, we had found higher expressions of HO-1 and NQO1 in the metformin-treated group when compared to those in the untreated group, which was significantly reversed by ML385 treatment (Figures 8(d)–8(f)). These findings have indicated that Nrf2 activation significantly induces the expression HO-1 and NQO1 under H_2_O_2_ condition and metformin further increases HO-1 and NQO1 expressions via activating the Akt/Nrf2/ARE pathway. Next, to verify that the protective effect of metformin was due to the amelioration of mitochondrial function, we detected the mitochondrial membrane potential (*Δψ*m) in PC12 cells using the JC-1 staining assay. Changes in the ratio of aggregate-to-monomer fluorescence were indicated as Δ*ψ*m. As shown in Figures 9(a) and 9(b), metformin treatment significantly increased the Δ*ψ*m of PC12 cells following exposure to H_2_O_2_ for 2 h when compared with those in the H_2_O_2_ group, ML385 group, and positive control group (CCCP group). These results revealed that metformin can restore the mitochondrial activity. The production of ROS is correlated with mitochondrial dysfunction and causes major adverse effect during secondary injury. Thus, we tried to understand whether metformin affects the level of intracellular ROS using a specific probe for hydrogen peroxide, 2′,7′-dichlorodihydrofluorescein diacetate (DCFH-DA). The fluorescence images had showed a markedly low density of fluorescence in the metformin group when compared with those in the H_2_O_2_ group, ML385 group, and ROSUP group (positive control group) (Figures 9(c) and 9(d)), indicating that metformin has a remarkable effect on the reduction of ROS. Additionally, we had further monitored the cellular ATP level. We found that the ATP levels were reduced after H_2_O_2_ and metformin treatment rescued it, whereas ML385 reversed the effect of metformin as shown by the ATP level (Figure 9(e)). Based on these results, metformin may restore mitochondrial dysfunction and then reduce the ROS level, resulting in neuron protection and regeneration.

## 4. Discussion

SCI is a serious neurological disease that can induce neurological dysfunction and permanent damage. Series of secondary injuries, including oxidative stress, mitochondrial dysfunction, and neuronal cell apoptosis, are considered as the major factor for disability [47]. Thus, new effective therapeutic treatments for reducing SCI-induced neurological disorders and tissue damages are urgently needed. Metformin, a glucose-lowering agent, is wildly used as the treatment of type II diabetes mellitus [48]. Our previous reports had also showed that metformin treatment improves functional recovery after SCI, in part by inhibiting the neuronal apoptosis and attenuating blood-spinal cord barrier disruption [32, 49]. However, it remains unclear whether metformin has a therapeutic effect in the recovery of axonal regeneration. In a present study, we found that metformin treatment significantly reduced spinal cord damage, decreased the neuronal apoptosis, inhibited oxidative stress, promoted axonal regeneration by stabilizing microtubules, and finally improved functional recovery after SCI in rat. The activation of the PI3K/Akt and Nrf2/ARE pathway were the potential mechanisms underlying metformin treatment for SCI.

As is known, injured axon has poor ability to regenerate after SCI [50]. Therefore, it is meaningful to explore the approaches to promote axonal regeneration after. Recently, various neuroregenerative researches have focused on dendritic and axonal repair to improve functional recovery after CNS injury [51]. Previous studies have shown that metformin exerts neuroprotective effect and promotes functional recovery of memory deficits via anti-inflammation and triggering neurogenesis [52]. In addition, recent researches have confirmed that metformin has a beneficial effect in promoting the nerve regeneration after peripheral nerve injury (PNI) [53]. These studies have suggested that metformin has a protective role in axonal regeneration. However, whether metformin has a therapeutic effect on axonal regeneration after SCI has not been reported. Based on these studies, we hypothesized that metformin can promote axonal regeneration after SCI. In our study, we found that metformin improved outgrowth of Ace-tubulin-labeled neurites, suggesting that metformin can promote axonal regeneration. Previous studies have demonstrated that remodeling of cytoskeleton structures, such as microtubules stabilization, is pivotal for the regrowth of injured axons and growth cone initiation [54]. We had found that metformin upregulated the expression of Ace-tubulin surrounding a lesion and increased the ratio of Ace-tubulin/Tyr-tubulin in primary cortical neurons under H_2_O_2_ condition, indicating that the effect of metformin on axonal regeneration was related to microtubule stabilization. Our present study has also revealed that the PI3K/Akt signaling pathway has neuroprotective effects on the central nervous system. Meanwhile, another study has further verified that the PI3K/Akt signaling pathway is involved in the protective effect of metformin on ischemic heart [35]. Moreover, PI3K/Akt have also played an important role in stabilizing microtubule structure to repair neurites after SCI [22]. These studies have demonstrated that metformin can activate the PI3K/Akt signaling pathway after SCI. Thus, we hypothesized that the PI3K/Akt pathway is essential for the effect of metformin on microtubule stabilization. In this study, we found that LY294002, a specific PI3K inhibitor, significantly reversed the effect of metformin on microtubule stabilization, suggesting that metformin has a potential to repair neurites by stabilizing microtubule structure after SCI.

Previous studies have showed that oxidative stress exerts a destructive role during SCI and diabetic neuropathy [55, 56]. Excessive oxidative stress with ROS accumulation can lead to neuronal apoptosis, which is not beneficial for nerve regeneration [57]. Many studies have reported that the Akt pathway plays an important role in the antiapoptosis process [58]. In this study, we found that metformin enhanced the number of Nissl bodies and maintained their normal morphology by activating the PI3K/Akt pathway, indicating that metformin treatment can protect neurons from SCI-induced apoptosis through the PI3K/Akt signaling pathway. In addition, Lu et al. had verified that fibroblast growth factor 21 improved functional recovery and axonal regeneration through regulating oxidative stress after PNI [59]. Reducing excessive oxidative stress improves the locomotor functional recovery after SCI [60]. One previous study has also demonstrated that metformin restores mitochondrial biogenesis by inhibiting of the PDK4/oxidative stress-mediated apoptosis pathway [61]. Based on these studies, we hypothesized that metformin has an important role in preventing excessive oxidative stress after SCI. To verify this hypothesis, we had exposed the PC12 cells to H_2_O_2_ to induce oxidative stress and treated it with 1 mM metformin. The results showed that massive accumulation of ROS was elicited in PC12 cells, which were markedly ameliorated with the treatment of metformin. Moreover, we had also found that metformin has a great antioxidative capability in vitro and in vivo, manifesting in marked increase levels of NQO1 and HO-1. In the antioxidant defensive system, Nrf2 is a pivotal antioxidative defender that binds to the ARE to maintain a normal oxidative level [62]. Moreover, many studies have shown that the Nrf2/ARE pathway plays an important role during oxidative stress [63]. Consistent with a prior study, our findings had also shown that the antioxidative capability of metformin was partially reversed by ML385 (a novel and specific Nrf2 inhibitor), suggesting that the Nrf2/ARE signaling pathway may be a potential mechanism underlying metformin protecting against oxidative stress after SCI.

It is well known that mitochondria are the principal power place of eukaryotic cell organelles and lead to ATP generation through the electron transport chain of oxidative phosphorylation reaction. Additionally, Singh et al. have demonstrated that mitochondrial dysfunction plays a significant role in secondary injury after neuronal injury, which induces the accumulation of ROS, neuronal cell death, and impairment of energy transduction [64]. On the other hand, axonal regeneration is a complex process and requires normal mitochondrial function in providing energy [65]. Thus, maintaining the normal mitochondrial function is crucial for axonal regeneration. Pintana et al. had reported that metformin can prevent brain mitochondrial dysfunction and restore learning behavior in high-fat diet-induced insulin-resistant rats [66]. Based on these studies, we hypothesized that metformin has a critical role in mitochondrial function in neuronal cells. We have found that metformin protected mitochondrial membrane potential and ATP levels from H_2_O_2_ condition in vitro. This result has indicated that the effect of metformin for SCI recovery is partly accomplished by protecting mitochondrial function.

Numerous studies have demonstrated that Akt exerts potent antioxidant effects via augmenting the transcriptional activity of Nrf2 [67]. Nrf2 is a key transcription factor that binds to the antioxidant response element (ARE) and then preserves a normal oxidative stress level [62]. In addition, Akt/Nrf2 signaling is regarded as the crucial molecular regulatory mechanism for alleviating oxidative stress-induced neuronal damage [68–70]. However, there is no clear evidence verified whether the role of metformin on antioxidative stress was closely related to Akt/Nrf2/ARE signaling after SCI. In this study, metformin markedly upregulated the expression levels of total Nrf2, nuclear Nrf2, NQO1, and HO-1 in vivo and in vitro. We had also found that metformin increased the expression of phosphorylation Akt. These results have indicated that the neuroprotective effect of metformin might be attributable to its antioxidant capacity through activating the Akt/Nrf2/ARE pathway.

Our study has identified that metformin exerts a great neuroprotection role after SCI and clarifies the related mechanisms underlying metformin treatment for SCI. However, there were several issues that need further research to be done. Firstly, as a more practical and less invasive route, oral administration of metformin is more beneficial for clinical application, but this might exhibit a different dose response curve when comparing with i.p. injection. Thus, it is necessary to further determine the efficacy of oral administration of metformin on the prevention of SCI. Secondly, it is well known that diabetes aggravates the prognosis of SCI [71], but the rats subjected to SCI were normal SD rats in our current study. Hence, as a traditional antidiabetic drug, the efficacy of metformin on SCI prevention in diabetic rats needs to be validated in the future study.

## 5. Conclusion

Our current study has demonstrated that metformin treatment significantly reduces spinal cord damage and subsequently improves the functional recovery after SCI. Additionally, we have firstly demonstrated that the protective effect of metformin on SCI is related to the reduction of neuronal cell apoptosis and the promotion of axonal regeneration by stabilizing microtubules. Moreover, suppressing excessive oxidative stress and restoring mitochondrial function are essential for the positive role of metformin with the involvement of the Akt/Nrf2/ARE signaling pathway. Our study suggested that metformin may be suitable as the potential therapeutic strategies for SCI recovery.

## Data Availability

The data used to support the findings of this study are available from the corresponding authors upon request.
